# Prevalence of and factors associated with Na + /K + imbalances in a population of children hospitalized with febrile urinary tract infection

**DOI:** 10.1007/s00431-024-05784-0

**Published:** 2024-10-02

**Authors:** Pierluigi Marzuillo, Stefano Guarino, Luigi Annicchiarico Petruzzelli, Milena Brugnara, Ciro Corrado, Anna Di Sessa, Gabrile Malgieri, Marco Pennesi, Floriana Scozzola, Francesca Taroni, Andrea Pasini, Claudio La Scola, Giovanni Montini, on behalf of CAKUT-UTI study group of the Italian Society of Pediatric Nephrology, Mattia Arenella, Salvatore Alfiero, Francesco Baccelli, Giulia Delcaro, Giulia Gallotta, Marcello Lanari, Maya Lorenzi, Emanuele Miraglia del Giudice, Carmine Pecoraro, Sara Picassi, Luca Pierantoni, Chiara Tosolini, Laura Venditto

**Affiliations:** 1 Department of Woman, Child and of General and Specialized Surgery, Università Degli Studi Della Campania “Luigi Vanvitelli”, Vico Luigi De Crecchio 2, 80138 Naples, Italy; 2 Pediatric Nephrology and Dialysis Unit, Santobono Children’s Hospital, Naples, Italy; 3 Pediatria C, Ospedale Donna Bambino, Verona, Italy; 4 Pediatric Nephrology, “G. Di Cristina” Hospital, Palermo, Italy; 5 Institute for Maternal and Child Health-IRCCS Burlo Garofolo, Trieste, Italy; 6 Pediatric Unit, Ca’ Foncello Civil Hospital, Treviso, Italy; 7 Pediatric Nephrology, Dialysis and Transplant Unit, Fondazione Ca’ Granda IRCCS, Policlinico Di Milano, Milan, Italy; 8 Pediatric Nephrology and Dialysis Unit, IRCCS Azienda Ospedaliero-Universitaria Di Bologna, Bologna, Italy; 9 Giuliana and Bernardo Caprotti Chair of Pediatrics, Department of Clinical Sciences and Community Health, University of Milan, Milan, Italy

**Keywords:** Transient secondary pseudo-hypoaldosteronism, Sodium, Potassium, Urinary tract infection, Congenital anomalies of the kidney and urinary tract, Children

## Abstract

We aimed to assess the prevalence of and factors associated with Na + /K + imbalances in children hospitalized for febrile urinary tract infection (fUTI). This retrospective Italian multicenter study included children aged 18 years or younger (median age = 0.5 years) who were discharged with a primary diagnosis of fUTI. Na + /K + imbalances were classified as hyponatremia (sodium < 135 mEq/L), hypernatremia (sodium > 145 mEq/L), hypokalemia (potassium < 3.5 mEq/L), hyperkalemia (potassium > 5.5 mEq/L), and concurrent hyponatremia and hyperkalemia, in the absence of evidence of hemolyzed blood samples. Among the 849 enrolled children, 23% had hyponatremia, 6.4% had hyperkalemia, 2.9% had concurrent hyponatremia and hyperkalemia, 0.7% had hypokalemia, and 0.4% had hypernatremia. In the multiple logistic regression analysis, after applying the Bonferroni correction, only C-reactive protein (C-RP) levels were significantly associated with hyponatremia (OR = 1.04; 95% CI: 1.02–1.06; *p* < 0.001), only age was significantly associated with hyperkalemia (OR = 1.7; 95% CI: 1.1–2.7; *p* = 0.01), and only CAKUT was significantly associated with concurrent hyponatremia and hyperkalemia (OR = 4.3; 95% CI: 1.7–10.8; *p* = 0.002). Even after adjusting for the presence of kidney hypoplasia, abnormal renal echogenicity, pelvi-caliceal dilation, ureteral dilation, uroepithelial thickening of the renal pelvis, bladder abnormalities, pathogen other than *E. coli*, concurrent hyponatremia and hyperkalemia persisted significantly associated with CAKUT (OR = 3.6; 95% CI: 1.2–10.9; *p* = 0.02).

*Conclusion*: Hyponatremia was the most common Na + /K + imbalance in children hospitalized for fUTI, followed by hyperkalemia and concurrent hyponatremia and hyperkalemia. C-RP levels were most strongly associated with hyponatremia, age with hyperkalemia, and CAKUT with concurrent hyponatremia and hyperkalemia (suggestive of transient secondary pseudo-hypoaldosteronism). Therefore, in children who develop concurrent hyponatremia and hyperkalemia during the course of a fUTI, an underlying CAKUT could be suspected.

**Table:** 

**What is known:** *• Na+ and K+ abnormalities can occur in patients hospitalized for febrile urinary tract infection (fUTI).* *• Concurrent hyponatremia and hyperkalemia during fUTI may suggest transient secondary pseudo-hypoaldosteronism (TPHA), for which limited data on prevalence are available.* **What is new:** *• The most common Na+/K+ imbalance in children hospitalized with fUTI was hyponatremia (23%), followed by hyperkalemia (6.4%), concurrent hyponatremia and hyperkalemia (2.9%), hypokalemia (0.7%), and hypernatremia (0.4%).* *• Concurrent hyponatremia and hyperkalemia were mainly associated with CAKUT, while hyponatremia alone correlated with high C-reactive protein and hyperkalemia alone with younger age. In cases of concurrent hyponatremia and hyperkalemia during fUTI, an underlying CAKUT should be suspected.*

**What is known:**

*• Na+ and K+ abnormalities can occur in patients hospitalized for febrile urinary tract infection (fUTI).*

*• Concurrent hyponatremia and hyperkalemia during fUTI may suggest transient secondary pseudo-hypoaldosteronism (TPHA), for which limited data on prevalence are available.*

**What is new:**

*• The most common Na+/K+ imbalance in children hospitalized with fUTI was hyponatremia (23%), followed by hyperkalemia (6.4%), concurrent hyponatremia and hyperkalemia (2.9%), hypokalemia (0.7%), and hypernatremia (0.4%).*

*• Concurrent hyponatremia and hyperkalemia were mainly associated with CAKUT, while hyponatremia alone correlated with high C-reactive protein and hyperkalemia alone with younger age. In cases of concurrent hyponatremia and hyperkalemia during fUTI, an underlying CAKUT should be suspected.*

## Introduction

Several reports indicate that abnormalities in Na + and K + can occur in patients hospitalized for febrile urinary tract infection (fUTI) [1–6].

The course of a fUTI can be complicated by hyponatremia, hyperkalemia, and acidosis, likely due to kidney tubular under-responsiveness to aldosterone. This condition is known as transient secondary pseudo-hypoaldosteronism (TPHA) [7–9]. The association between fUTI and TPHA has mainly been reported in case reports or small case series [7, 10–19].

While TPHA during fUTI is considered rare, there is limited data on its prevalence during fUTI [1, 6].

No study has systematically investigated the factors associated with Na + /K + imbalances during the course of a fUTI. Therefore, our aim was to evaluate the prevalence of Na + /K + imbalances, in the absence of hemolysis, in a population of children hospitalized for fUTI [20]. Furthermore, we aimed to delineate the clinical characteristics of patients presenting with these imbalances and identify the associated factors.

## Methods

As previously described [20], we collected data from all children discharged with the primary diagnosis of fUTI from January 1, 2017, to December 31, 2021. Patients aged between birth and 18 years and with the availability of serum creatinine levels (measured by isotope dilution mass spectrometry (IDMS)-traceable method), kidney ultrasound (and if performed, voiding cystourethrography findings), serum electrolytes, blood count, and markers of inflammation were enrolled. All the listed parameters were available for all the enrolled patients, with the exception of procalcitonin, a marker of inflammation, which was available in a subgroup of 390 patients. Data from biochemical samples were recorded from samples collected at admission or at a second blood sample if hemolysis was present [8]. None of the patients with hyponatremia received hypotonic intravenous fluids.

Na + /K + imbalances were evaluated in the absence of evidence of hemolyzed blood samples, ambiguous genitalia, and in the presence of normal neonatal screening for congenital adrenal hyperplasia, as well as electrolyte normalization without the administration of hydrocortisone or fludrocortisone but solely through antibiotic therapy and saline infusion.

The study was approved by the Research Ethics Committee of University of Campania (approval no. 12770/2020).

### Definitions of Na + /K + imbalances

1. ***Hyponatremia***
  Hyponatremia was defined by serum sodium levels < 135 mEq/L [21].
2. ***Hypernatremia***
  Hypernatremia was defined by serum sodium levels > 145 mEq/L [21].
3. ***Hypokalemia***
  Hypokalemia was defined by serum potassium levels < 3.5 mEq/L [21].
4. ***Hyperkalemia***
  Hyperkalemia was defined by serum potassium levels > 5.5 mEq/L [21].
5. ***Hyponatremia and hyperkalemia***
  This condition was defined by serum sodium levels < 135 mEq/L and serum potassium levels > 5.5 mEq/L [8]. We chose to analyze patients with concurrent hyponatremia and hyperkalemia separately because this imbalance is indicative of TPHA [8]. Besides the initial data collection, we reviewed the clinical charts of patients with this Na + /K + imbalance to gather additional data on serum aldosterone and serum bicarbonate, where available [8].

### fUTI diagnosis

fUTI was defined by the presence of urinary leukocytes with or without nitrites, positive urine culture (single microorganism), and fever > 38 °C without other symptoms [20, 22, 23].

### Acute kidney injury

Acute kidney injury (AKI) was defined by the serum creatinine criterion indicated by the Kidney Disease/Improving Global Outcomes (KDIGO) [24]. When basal creatinine was unknown, an estimated basal serum creatinine was calculated [20]. This method, compared with the utilization of measured basal serum creatinine, was validated in the age range of our population [20].

### Statistical analysis

Continuous variables were analyzed by the independent-sample *t*-test in case of normal distribution and the Mann–Whitney test in case of non-normal distribution. Qualitative variables were compared by the chi-square or Fisher exact tests, as appropriate. The length of stay was examined through survival analysis using the Kaplan–Meier method. The day of admission was considered the starting point, while the endpoint was the date of discharge. Kaplan–Meier curves were compared by log-rank test.

#### Logistic regression models to analyze factors associated with Na + /K + imbalances

Due to the very low number of patients with hypernatremia or hypokalemia, factors associated with these conditions were not analyzed. Instead, logistic regression models were employed to explore associations with hyponatremia, hyperkalemia, and their combination. After comparing the characteristics of patients with and without these imbalances (Table 1), parameters associated (*p* < 0.05) with such imbalances were included in the univariate logistic regression analysis. Electrolytes were not included in the univariate logistic regression analysis as they were used for patient classification.

**Table 1:** Clinical and laboratory characteristics of children hospitalized for fUTI with and without Na + /K + imbalances

	Hyponatremia			Hyperkalemia			Hyponatremia and hyperkalemia		
	No	Yes	*p*	No	Yes	*p*	No	Yes	*p*
No	654	195	–	795	54	–	824	25	–
Age, yr, median (IQR)	0.46 (1.5)	0.88 (2.7)	< 0.001	0.58 (2.0)	0.23 (0.32)	< 0.001	0.55 (1.8)	0.4 (0.94)	0.20
Female sex, no (%)	283 (43.3)	92 (47.2)	0.33	358 (45.0)	17 (31.5)	0.05	369 (44.8)	6 (24)	0.04
Birth weight < 2.5 kg, no (%)	49 (7.5)	13 (6.7)	0.70	55 (6.9)	7 (13.0)	0.10	59 (7.2)	3 (12.0)	0.36
Preterm birth, no (%)	103 (15.7)	27 (13.8)	0.52	114 (14.3)	16 (29.6)	0.003	126 (15.3)	4 (16)	0.93
Duration of symptoms before admission days, median (IQR)	1.0 (2.0)	2.0 (2.0)	0.16	1.0 (2.0)	2.0 (2.0)	0.76	1.0 (2.0)	1.5 (2.0)	0.97
CKD, no (%)	28 (4.3)	7 (3.6)	0.67	34 (4.3)	1 (1.9)	0.39	33 (4)	2 (8)	0.32
AKI, no (%)	87 (13.3)	37 (19.0)	0.049	120 (15.1)	4 (7.4)	0.16	117 (5.9)	7 (28)	0.05
Presence of CAKUT, no (%)	219 (33.5)	66 (33.8)	0.93	273 (34.3)	12 (22.2)	0.07	267 (31.4)	18 (72)	< 0.001
Bilateral CAKUT*, no (%)	77 (11.8)	13 (6.7)	0.06	85 (10.7)	5 (9.2)	0.91	80 (9.7)	10 (40)	< 0.001
Vesicoureteral reflux, no (%)	157 (24.0)	46 (23.6)	0.90	195 (24.5)	8 (14.8)	0.10	189 (22.9)	14 (56)	< 0.001
Primary megaureter, no (%)	29 (4.4)	3 (1.5)	0.10	30 (3.8)	2 (3.7)	0.99	24 (2.9)	8 (32)	< 0.001
PUV, no (%)	8 (1.2)	2 (1.0)	0.99	9 (1.1)	1 (1.8)	0.48	9 (1.1)	1 (4)	0.18
UPJO, no (%)	15 (2.3)	3 (1.6)	0.78	18 (2.3)	0 (0)	0.62	15 (2.1)	3 (12)	0.02
Uno- or bilateral renal hypoplasia, no. (%)	54 (8.3)	25 (12.8)	0.05	75 (9.4)	4 (7.4)	0.62	77 (9.2)	2 (8)	0.99
Bladder abnormalities, no. (%)	14 (2.1)	1 (0.5)	0.21	14 (1.8)	1 (1.9)	0.96	14 (1.7)	1 (4)	0.36
Vomiting, no (%)	122 (18.7)	45 (23.1)	0.18	162 (20.4)	5 (9.3)	0.05	159 (19.3)	8 (32.0)	0.12
Na, mEq/L, mean (SDS)	137.4 (2.6)	132.7 (1.7)	< 0.001	136.2 (3.1)	138.2 (2.3)	< 0.001	136.4 (2.9)	130.6 (2.4)	< 0.001
K, mEq/L, mean (SDS)	4.8 (0.7)	4.5 (0.6)	< 0.001	4.6 (0.6)	5.8 (0.3)	< 0.001	4.7 (0.6)	6.0 (0.7)	< 0.001
Cl, mEq/L, median (IQR)	102.0 (4.9)	97.0 (5.0)	< 0.001	101.0 (5.0)	102.0 (4.0)	0.04	101.0 (5.0)	97.0 (11.0)	0.04
Maximal body temperature, °C, mean (SDS)	38.7 (0.90)	39.0 (0.96)	0.001	38.8 (0.96)	38.8 (0.95)	0.17	38.8 (1.0)	38.5 (0.8)	0.08
Refill > 2 s, no (%)	25 (3.8)	6 (3.1)	0.63	31 (3.9)	0 (0)	0.25	29 (3.5)	2 (8.0)	0.24
HR > 2SDS for age, no (%)	124 (19.0)	44 (22.6)	0.27	161 (20.3)	7 (13.0)	0.19	161 (19.5)	7 (28)	0.29
Need of bolus, no (%)	24 (3.7)	3 (1.5)	0.17	26 (3.3)	1 (1.9)	0.99	24 (2.9)	3 (12)	0.01
WBC, n/mcL, median (IQR)	14,960 (8275)	16,205 (8120)	0.08	15,360 (8100)	15,490 (6840)	0.99	15,380 (8240)	16,290 (6660)	0.76
Neutrophils, n/mcL, median (IQR)	8005 (6698)	9660 (6423)	0.001	8600 (6980)	6650 (5560)	0.10	8495 (6860)	7990 (7870)	0.76
Platelets, n/mcL, median (IQR)	392,500 (200,250)	439,500 (162,000)	0.005	375,000 (193,000)	443,000 (212,000)	0.01	380,000 (200,000)	435,000 (295,000)	0.05
C-RP, mg/dL, mean (SDS)	8.1 (8.1)	11.7 (9.0)	< 0.001	9.1 (8.5)	6.8 (8.5)	0.07	8.9 (8.2)	11.7 (9.5)	0.1
Length of stay, days, median (IQR)	5.0 (4.0)	5.0 (3.0)	0.98	5.0 (3.0)	6.0 (4.0)	0.10	5.0 (4.0)	7.0 (6.0)	0.04
Procalcitonin, ng/mL, mean (SDS)	6.5 (1.6)	14.0 (3.1)	< 0.001	8.6 (2.2)	4.4 (1.1)	0.30	8.4 (1.2)	8.6 (1.9)	0.97
Pathogen other than *E. coli*, no. (%)	127 (19.4)	33 (16.9)	0.43	150 (18.9)	10 (18.5)	0.95	150 (18.2)	10 (40)	0.006

^*^This data refers to the 285 patients with uropathies

For normal distributed variables means ± standard deviation scores are shown, while for non-parametric ones, median and interquartile range are shown

Abbreviations: *AKI* acute kidney injury, *CAKUT* congenital anomalies of the kidney and urinary tract, *CKD* chronic kidney disease, *C-RP* C-reactive protein, *IQR* interquartile range, *Na* sodium, *K* potassium, *HR* heart rate, *SDS* standard deviation score, *WBC* white blood cell count

Factors with a *p* < 0.05 in univariate logistic regressions were included in multiple logistic regressions. Significance in multiple logistic regression analyses was determined using the Bonferroni correction.

When analyzing factors associated with hyponatremia, although neutrophils were significantly associated with hyponatremia (*p* < 0.05) (Table 1), they were not included in the univariate logistic regression analysis due to high collinearity with C-reactive protein (C-RP) and potential influence from patient age [25, 26]. Procalcitonin was also excluded due to high collinearity with C-RP and its availability only in a subgroup of patients.

When analyzing factors associated with hyponatremia and hyperkalemia, bilateral congenital anomalies of the kidney and urinary tract (CAKUT) and a single type of CAKUT were excluded from the model due to their high collinearity with CAKUT.

#### Logistic regression model to further test the association between CAKUT and concurrent hyponatremia and hyperkalemia

Additionally, we assessed the association between CAKUT and concurrent hyponatremia and hyperkalemia in a separate multiple logistic regression analysis. This analysis adjusted for diagnostic covariates typically used to evaluate the risk of vesicoureteral reflux (VUR) in the context of a fUTI episode, including unilateral or bilateral kidney hypoplasia, abnormal renal echogenicity, pelvi-caliceal dilation, ureteral dilation, uroepithelial thickening of the renal pelvis, bladder abnormalities, and pathogen other than *E. coli* [23].

## Results

### General characteristics

The study population comprised 849 children hospitalized for fUTI with available serum Na + and K + levels. Of these, 375 (44.2%) were female, and the median age was 0.5 years (range 0–18 years) [20]. One hundred and twenty-four out of 849 patients presented with AKI [20].

An imbalance in serum Na + or K + levels was found in a total of 283 patients (33.3%). Specifically, hyponatremia alone was found in 195 out of 849 children (23.0%), hypernatremia alone in 3 patients (0.4%), hypokalemia alone in 6 patients (0.7%), and hyperkalemia alone in 54 patients (6.4%). The combination of hyponatremia and hyperkalemia was present in 25 out of 849 children (2.9%). For these 25 patients, aldosterone levels (all > 100 mg/dL) were available for 9 patients, and bicarbonate levels were available for 11 patients, with 9 of these showing values < 20 mmol/L.

In all cases, the Na + /K + imbalance was resolved only with antibiotic treatment and saline infusion.

As per the definition, none of the patients with the Na + /K + imbalance exhibited other serum abnormalities indicative of hemolyzed blood samples. Additionally, no female patients with this imbalance presented with ambiguous genitalia or clitoral enlargement, and no male patients presented with phallic enlargement and scrotal hyperpigmentation. The neonatal screening also excluded salt-losing congenital adrenal hyperplasia in our cohort.

Patients with hyponatremia alone were older and had higher maximal body temperature, neutrophils, platelets, C-RP, procalcitonin levels, and a higher prevalence of AKI compared to those without hyponatremia. Patients with hyperkalemia alone were younger, had higher platelet levels, and higher prevalence of preterm birth compared with patients without hyperkalemia. Patients with both hyponatremia and hyperkalemia had a higher prevalence of male sex, AKI, CAKUT, bilateral CAKUT, vesicoureteral reflux, primary megaureter, ureteropelvic junction obstruction, the need of bolus, pathogen other than *E. coli*, and higher platelets levels and length of stay compared with patients without hyponatremia and hyperkalemia (Table 1).

**Fig. 1:**
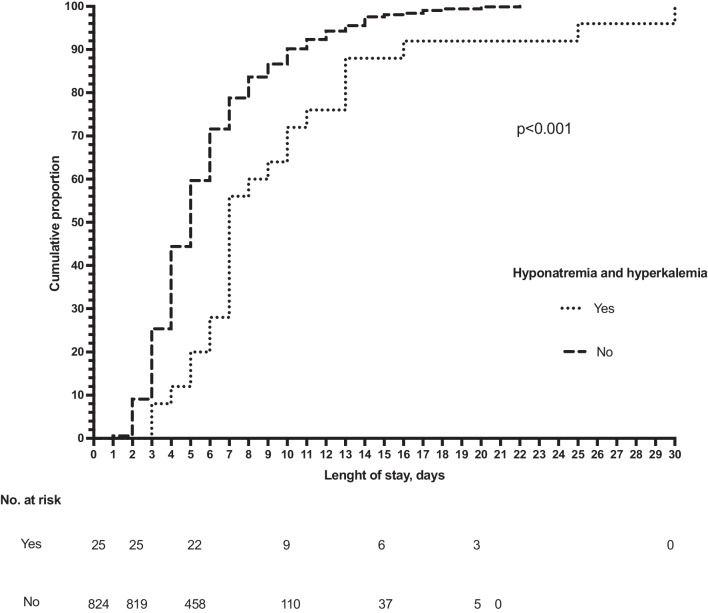
Length of stay evaluated by Kaplan–Meier analysis comparing patients with and without concurrent hyponatremia and hyperkalemia suggesting TPHA. The cumulative proportion of discharge of patients without Na + /K + imbalance was 9.1% at 2 days, 44.4% at 5 days, 86.6% at 10 days, 97.6% at 15 days, 99.8% at 20 days, and 100% at 21 days. For the patients with Na + /K + imbalance, the cumulative proportion of discharge was 0% at 2 days, 20.0% at 5 days, 72% at 10 days, 88% at 15 days, 92% at 20 days, and 100% at 30 days. Log-rank test comparing the three Kaplan–Meier curves showed a *p* < 0.001

A longer length of stay for patients with compared with those without concurrent hyponatremia and hyperkalemia was also confirmed by Kaplan–Meier analysis (Fig. 1).

### Logistic regression analyses

In the multiple logistic regression analysis, after applying the Bonferroni correction, only C-RP levels remained significantly associated with hyponatremia (Table 2), only age remained significantly associated with hyperkalemia (Table 3), and only CAKUT remained significantly associated with concurrent hyponatremia and hyperkalemia (Table 4).

**Table 2:** Exploratory analysis of prognostic factors potentially associated with hyponatremia alone in children hospitalized with fUTI

	Univariate			Multiple		
	OR	95% CI	*p*	OR	95% CI	*p*^#^
Age*	1.05	0.96–1.06	0.83	–	–	–
AKI	1.5	0.98–2.3	0.06	–	–	–
Maximal body temperature	1.3	1.1–1.6	0.001	1.2	0.96–1.4	0.11
Platelets**	1.1	1.05–1.25	0.036	1.08	0.96–1.2	0.22
C-RP***	1.05	1.03–1.06	< 0.001	1.04	1.02–1.06	< 0.001

Abbreviations: *AKI* acute kidney injury, *CI* confidence interval, *C-RP* c-reactive protein, *fUTI* febrile urinary tract infection, *OR* odds ratio

^#^*p* threshold after Bonferroni correction was 0.017

^*^for each increase of 0.1 years

^**^for each increase of 100,000 platelets/mcL

^***^for each increase of 1 mg/dL

**Table 3:** Exploratory analysis of prognostic factors potentially associated with hyperkalemia alone in children hospitalized with fUTI

	Univariate			Multiple		
	OR	95% CI	*p*	OR	95% CI	*p*^#^
Age*	1.9	1.2–3.0	< 0.001	1.7	1.1–2.7	0.01
Preterm birth	2.5	1.4–4.7	< 0.001	2.0	1.08–3.8	0.028
Platelets**	1.3	1.1–1.6	< 0.001	1.2	1.0–1.4	0.06

Abbreviations: *CI* confidence interval, *fUTI* febrile urinary tract infection, *OR* odds ratio

^#^*p* threshold after Bonferroni correction was 0.017

^*^for each decrease of 0.1 years

^**^for each increase of 100,000 platelets/mcL

**Table 4:** Exploratory analysis of prognostic factors potentially associated with concurrent hyponatremia and hyperkalemia (suggestive of TPHA) in children hospitalized with fUTI

	Univariate			Multiple		
	OR	95% CI	*p*	OR	95% CI	*p*^#^
Male sex	2.6	1.01–6.5	0.046	2.4	0.9–6.1	0.07
Presence of CAKUT	5.4	2.2–13.0	< 0.001	4.3	1.7–10.8	0.002
Need of bolus	4.5	1.3–16.2	0.02	4.6	1.2–17.2	0.02
Pathogen other than *E. coli*	3.0	1.3–6.8	0.009	1.8	0.8–4.4	0.17

Abbreviations: *CAKUT* congenital anomalies of the kidney and urinary tract, *CI* confidence interval, *fUTI* febrile urinary tract infection, *OR* odds ratio, *TPHA* transient secondary pseudo-hypoaldosteronism

^#^*p* threshold after Bonferroni correction was 0.012

Finally, concurrent hyponatremia and hyperkalemia remained significantly associated with CAKUT (OR = 3.6; 95% CI: 1.2–10.9; *p* = 0.02), even after adjusting for the presence of unilateral or bilateral kidney hypoplasia, abnormal renal echogenicity, pelvi-caliceal dilation, ureteral dilation, uroepithelial thickening of the renal pelvis, bladder abnormalities, pathogen other than *E. coli*.

## Discussion

Studies involving between 24 and 313 children with fUTI reported hyponatremia in more than 50% of the cases, hyperkalemia in 10–25%, hypernatremia in 5–10%, and hypokalemia in 5–10% of the cases [1–6, 8]. Our study involves a large, nationwide Italian population of 849 children with fUTI, aiming to describe the prevalence of and factors associated with Na + /K + imbalances in the absence of hemolysis, ambiguous genitalia, congenital adrenal hyperplasia, and with electrolyte normalization without the administration of hydrocortisone or fludrocortisone. We identified Na + /K + imbalances in 33.3% of the participants. Consistent with previous reports, we found that the most common abnormality was hyponatremia (23%), followed by hyperkalemia (6.4%), hypokalemia (0.7%), and hypernatremia (0.4%). The discrepancy in the absolute prevalence of Na + /K + imbalances across studies could be due to the wide variability in the range of enrolled patients.

When concurrent hyponatremia and hyperkalemia are observed during the course of a fUTI, TPHA may be suspected. Data on TPHA prevalence during the course of fUTI are limited. The associations described in the literature mainly derive from case reports or small case series [7, 10–19]. Previously, Gil-Ruiz et al. examined a population of 113 prospectively enrolled children with fUTI and found a prevalence of hyperkalemia with a transtubular potassium gradient values (TTKG) < 5 (indicating an inappropriate response to hyperkalemia and indirectly suggesting TPHA) of 11.5% [6]. On the other hand, an old retrospective study by Sperl et al., including approximately 300 children with APN, showed biochemical signs of aldosterone resistance (hyperkalemia and hyponatremia) in nearly 3% of the patients [1].

In our study, we found that the prevalence of concurrent hyponatremia and hyperkalemia, which may potentially suggest TPHA, was approximately 3%, similar to the study by Sperl et al. [1]. The differences found with the study by Gil-Ruiz et al. [6] could be linked to the fact that they considered only the presence of hyperkalemia with TTKG < 5 and not the concomitant hyponatremia to suspect TPHA. In neither of the previous studies were aldosterone levels available [1, 6]. In our study cohort, aldosterone levels were available in 9 patients, and, in all patients, they were > 100 ng/dL.

Information from case reports or case series indicates that TPHA can be linked to obstructive uropathies and/or fUTI [7, 10–19], with the latter not necessarily linked to the presence of an underlying CAKUT [6]. Our report is the first to systematically investigate factors associated with concurrent hyponatremia and hyperkalemia, potentially suggesting TPHA. In line with the findings of the systematic review by Betti C et al. [27], we added to the current knowledge that this mainly occurs in male patients and is more prevalent among patients with CAKUT underlying fUTI, AKI, the need for bolus, higher platelets levels, and fUTI secondary to pathogen other than *E. coli*. Additionally, patients developing the Na + /K + imbalance presented with a longer hospital stay.

The fact, that concurrent hyponatremia and hyperkalemia mainly involved male patients, however, could be an epiphenomenon due to higher incidence of CAKUT in males.

In multiple logistic regression analysis, we found that the presence of CAKUT underlying fUTI was the factor most strongly associated with the development of the Na + /K + imbalance suggestive of TPHA (hyponatremia and hyperkalemia), with a fourfold increased risk of this imbalance in cases of CAKUT underlying fUTI. To reinforce this finding, we also observed that this association remained significant even after adjusting for the diagnostic covariates typically used to evaluate the risk of VUR underlying an fUTI episode [22, 23]. Specifically, in the case of concurrent hyponatremia and hyperkalemia during fUTI, the risk of CAKUT increased by 3.6 times.

On the other hand, evaluating factors associated with the other Na + /K + imbalances, we found that hyponatremia was mainly associated with C-RP levels (Table 2) confirming that hyponatremia alone during the course of fUTI could be linked to systemic inflammation [3, 28]. Additionally, we found that hyperkalemia was primarily associated with younger age (Table 3). Although we carefully monitored for signs of hemolysis in the blood samples of the enrolled patients, minimal hemolysis may still have occurred in younger children, which could explain the observed association between hyperkalemia and age. Nonetheless, we believe it is important to emphasize that neither hyponatremia nor hyperkalemia alone was associated with CAKUT. This suggests that only the combination of hyponatremia and hyperkalemia during the course of a fUTI should raise suspicion of an underlying CAKUT.

Elevated platelet levels were associated with all electrolyte abnormalities analyzed. We believe this may be an epiphenomenon related to increased inflammation [29] or age [30]. Indeed, when platelet levels were included in the multivariate logistic regression, their significant association with hyponatremia and hyperkalemia was no longer evident.

A limitation of our study is its retrospective design, which did not allow for the measurement of aldosterone serum levels in all patients and then we can only give an idea of the prevalence of a Na + /K + imbalance suggestive of TPHA. Moreover, in children aged more than 12 months, a less severe form of TPHA can occur. In these cases, Na + wasting without hyponatremia, hyperkalemia, or acidosis can be present [31]. In our cohort, these cases have not been diagnosed because serum aldosterone levels were available only in a minor quote of patients with Na + /K + imbalance. On the other hand, even though we lack aldosterone levels for all patients with Na + /K + imbalance, in our 25 cases, the absence of hemolysis, neonatal screening excluding salt-losing congenital adrenal hyperplasia, and the normalization of the Na + /K + imbalance without intravenous hydrocortisone or fludrocortisone, but solely through antibiotic therapy and saline infusion, rule out other forms of hypoaldosteronism and strongly suggest a diagnosis of TPHA [8].

A strength of our study is the large sample of enrolled patients and the availability of accurate information about possible underlying CAKUT in patients selected according to the same recommendations [22, 23].

In conclusion, the most common Na + /K + imbalance was hyponatremia (23%), followed by hyperkalemia (6.4%), concurrent hyponatremia and hyperkalemia (2.9%), hypokalemia (0.7%), and hypernatremia (0.4%). Concurrent hyponatremia and hyperkalemia (suggestive of TPHA) may lead to a longer hospital stay. The factor most strongly associated with this imbalance during fUTI was CAKUT. Therefore, in children who develop concurrent hyponatremia and hyperkalemia during the course of a fUTI, an underlying CAKUT could be suspected.

On the other hand, hyponatremia alone was primarily associated with elevated C-RP levels, while hyperkalemia alone was linked to younger age.

Future prospective studies are needed to assess the prevalence of TPHA by measuring serum aldosterone levels in all patients, to confirm the data shown in this paper, and to investigate the potential presence of subclinical TPHA, as observed in patient with obstructive uropathies [32, 33].

## Data Availability

The datasets generated during and/or analysed during the current study are available from the corresponding author on request.

## Abbreviations

AKI: Acute kidney injury
CAKUT: Congenital anomalies of the kidney and urinary tract
C-RP: C-reactive protein
fUTI: Febrile urinary tract infection
KDIGO: Kidney Disease/Improving Global Outcomes
OR: Odds ratio
TPHA: Transient secondary pseudo-hypoaldosteronism
TTKG: Transtubular potassium gradient values
VUR: Vesicoureteral reflux
